# Genetic Architecture Underpinning Yield Components and Seed Mineral–Nutrients in Sesame

**DOI:** 10.3390/genes11101221

**Published:** 2020-10-18

**Authors:** Naama Teboul, Yaron Gadri, Zipi Berkovich, Ram Reifen, Zvi Peleg

**Affiliations:** 1 The Robert H. Smith Institute of Plant Sciences and Genetics in Agriculture, The Hebrew University of Jerusalem, Rehovot 7610001, Israel; naama.teboul@mail.huji.ac.il (N.T.); yaron.gadri@mail.huji.ac.il (Y.G.); 2 Institute of Biochemistry, Food Science and Nutrition, The Hebrew University of Jerusalem, Rehovot 7610001, Israel; berkovich.zipi@gmail.com (Z.B.); ram.reifen@mail.huji.ac.il (R.R.)

**Keywords:** bioavailability, biofortification, iron, QTL co-localization, macronutrients, micronutrient, zinc

## Abstract

Genetic dissection of yield components and seed mineral-nutrient is crucial for understanding plant physiological and biochemical processes and alleviate nutrient malnutrition. Sesame (*Sesamum indicum* L.) is an orphan crop that harbors rich allelic repertoire for seed mineral–nutrients. Here, we harness this wide diversity to study the genetic architecture of yield components and seed mineral–nutrients using a core-collection of worldwide genotypes and segregating mapping population. We also tested the association between these traits and the effect of seed nutrients concentration on their bio-accessibility. Wide genetic diversity for yield components and seed mineral–nutrients was found among the core-collection. A high-density linkage map consisting of 19,309 markers was constructed and used for genetic mapping of 84 QTL associated with yield components and 50 QTL for seed minerals. To the best of our knowledge, this is the first report on mineral–nutrients QTL in sesame. Genomic regions with a cluster of overlapping QTL for several morphological and nutritional traits were identified and considered as genomic hotspots. Candidate gene analysis revealed potential functional associations between QTL and corresponding genes, which offers unique opportunities for synchronous improvement of mineral–nutrients. Our findings shed-light on the genetic architecture of yield components, seed mineral–nutrients and their inter- and intra- relationships, which may facilitate future breeding efforts to develop bio-fortified sesame cultivars.

## 1. Introduction

Mineral elements play essential roles in almost every aspect of plants development, cellular, biochemical and physiological processes. In grain-crops, minerals are stored mainly in the seeds and affect germination and early seedling establishment. Mineral–nutrients are also essential for animal and human welfare, and their deficiencies are a widespread problem, known as the “hidden hunger”. More than two billion people across the world, mostly in low- and middle- income countries, suffer from micronutrient malnutrition as consequence of a single crop-based diet. Dietary micronutrient deficiencies, impair human health and development and expressed in various symptoms, including anemia, susceptibility to infectious diseases, blindness, impaired physical and cognitive development, growth retardation, depressed immune system and higher mortality rates [1,2]. Accessibility of sufficient amounts of nutrients in the human diet depends primarily on their composition in plants (especially seeds). Improving mineral–nutrients content in plant-based food is an imminent priority facing agricultural research. Enhancement of seed nutrients (i.e., bio-fortification), either through agronomic practices (soil or foliar fertilization) or genetically (breeding and biotechnology) offers a cost-effective, long-term and sustainable solution to alleviate malnutrition and related health problems (reviewed by [3]).

While enhancement of seed mineral-nutrient content is important, nutrient bio-accessibility (i.e., dietary intake of a nutrient that is absorbed and available to physiological functions) is a key factor for nutrient intake in plant-based diets. Bio-accessibility mainly depends on the chemical form and amount consumed, digestion, interaction between nutrients and organic compounds, and absorption in the gastrointestinal tract [4]. For example, phosphorus (P) is stored in the seeds mainly as phytic acid (or phytate), which is known to have an adverse effect on nutrient bio-accessibility, and considered as anti-nutrient as consequence of the chelation with cationic minerals such as zinc (Zn^2+^), iron (Fe^2+^ or Fe^3+^), potassium (K^+^), magnesium (Mg^2+^), calcium (Ca^2+^), and manganese (Mn^2+^) [5,6]. Hence, understanding the genetic architecture of seed minerals, and their genetic and phenotypic associations with one another and with other agronomic traits, will promote breeding efforts to enhance nutrient content and increase their bioavailability.

Sesame (*Sesamum indicum* L.; genome 2n = 2x = 26), which belongs to the Pedaliaceae family, is an important oilseed-crop worldwide. It is an erect to semi-erect indeterminate annual plant with a simple or branching rigid stem. The stem shape is round or square, with ovate to lanceolate leaves and leaf margins ranging from entire to serrate. Sesame growth period commonly ranges from 12–16 w, in which flowering begins about 30–40 d after sowing and blooming continues until maturity. Its yield components include number of plants per unit area, number of branches per plant, number of capsules per leaf axil, seeds per capsule and seed weight. Complex tradeoffs between yield component traits, significantly affect the final seed yield [7]. Indeterminate plant growth habit and seed shattering at maturity led to poor adaptation to modern farming techniques, such as mechanical harvesting) [8]. Currently, sesame is cultivated using traditional practices, primarily in tropical and subtropical regions of Asia, Africa, and South America.

Sesame seeds are used in a variety of food industries, such as oil production, cooking and baking, and in the pharmaceutical industry (reviewed by [9]). Seed oil and protein content showed a wide variation in sesame ranging from 33–58% and 14–30%, respectively [10]. Sesame has a high quality seed oil which makes it the “queen of oilseeds”, and is rich in unsaturated (UFA), polyunsaturated fatty acids (PUFAs), and antioxidants, such as sesamol, sesamin, sesamolin and sesaminol [11,12]. Sesame seeds are also traditionally known as a “health-food” due to their high nutritional values (e.g., iron, calcium, iodine and zinc; [13,14] and vitamins (e.g., thiamin, riboflavin, niacin, folic acid, vitamin E and B6; reviewed by [15]).

Wide genetic diversity for various morphological and physiological traits was found in the sesame genepool [16,17,18]. Here we harness this wide diversity to uncover the genetic architecture of yield components and seed mineral–nutrients in sesame. The current study aimed to (*i*) characterize the genetic diversity for yield components and seed quality traits in core-collection of worldwide sesame genotypes, (*ii*) determine the chromosomal locations and phenotypic effects of QTL associated with sesame yield components and seed quality, and (*iii*) study the phenotypic and genotypic association between the various traits and their effect of nutrient bio-accessibility. Our findings shed light on sesame genetic architecture of yield components and seed quality as well as their interactions, which serve as a basis for future breeding programs aiming to develop bio-fortified sesame cultivars.

## 2. Materials and Methods

### 2.1. Plant Material and Growth Conditions

A core-collection of 30 sesame genotypes was establish from our large sesame collection, based on geographic passport data, to represent various environmental conditions (Table S1). All genotypes had an indeterminate growth habit and a dehiscent capsule phenotype, except S-343 (determinate growth) and S-91 (indehiscent capsule). A complete randomized, one way, block design with 30 genotypes was carried out in an open field at the experimental farm of the Hebrew University of Jerusalem in Rehovot, Israel (34°47′ N, 31°54′ E; 54 m above sea level). The soil at this location is brown-red degrading sandy loam (Rhodoxeralf, American Soil Science Society classification) composed of 76% sand, 8% silt and 16% clay. The plots were drip irrigated up to field capacity according to the protocol developed in Gadri et al. [7]. Plants were treated with pesticides to avoid pathogens or insect pests and weeded manually once a week. Each plot consisted four rows of six plants (a total of 24 plants) spaced 20 cm between plants, with five replicates (total of 150 plots).

A mapping population was developed from a cross between S-91 (♀) and S-297 (♂) to obtain F_1_ seeds, followed by self-fertilization to obtain F_2_ seeds. S-91 was selected based on high mineral–nutrients composition and indehiscent capsule, and S-297 is widely used in our lab as a high-yield cultivar. The F_2_ mapping population, consisting of 149 plants, was grown in an insect-proof screen-house (0.27 × 0.78 mm pore size), together with five plants of each parental line. The soil was covered with a black weed mat to avoid weeds.

For bio-accessibility assay, a complete randomized block design, with two genotypes (S-91 and S-297), four replicates each, was carried out at the experimental farm of the Hebrew University of Jerusalem. Each plot consisted one row of six plants with a spacing of 15 cm between plants.

### 2.2. Phenotypic Characterization

Phenological traits: Flowering date (FD), was calculated for F_2_ population as the number of days from planting till first flower, and for the core-collection from planting till flowering of 50% of the plants in the plot. First flowering node (FN), number of nodes (NN), internode length (IL), plant height (PH) and number of secondary branches (SB) were measured either for individual F_2_ plant or as means of five plants from each plot. When plants on a plot reached physiological maturity (i.e., first capsule change color from green to yellow), plants from the middle rows were harvested, oven-dried (42oC for 48 h) and weighted. Number of capsules per plant (NCPP) and number of seeds per plant (SPP) were measured for core-collection. Capsule dehiscence was characterized, at maturity, using an index (CDI) ranging from one (indehiscent) to five (dehiscent). Capsule width (CW) and length (CL) were measured with a digital caliper, in seven replicates from each plant. Thousand seed weight (TSW) was calculated from the total seed weight. A sub-sample of 50 seeds for each line were scanned (HP Scanjet G2710) and images were analyzed by GrainScan software [19], to obtain the seed length (SL), width (SW), perimeter (SP), and area (SA). Seed color (SC) was calculated as the mean of obtained red (R), green (G), and blue (B) values.

### 2.3. Seed Nutrient Concentration Analysis

Seed micronutrients (zinc, Zn; iron, Fe; copper, Cu; and manganese, Mn) and macronutrients (calcium, Ca; magnesium, Mg; potassium, K; phosphorus, P; and sulfur, S) concentration were determined by a radial Inductively Coupled Plasma Atomic Emission Spectrometer (ICP-AES, ARCOS, Spectro Analytical Instruments GmbH, Kleve, Germany), equipped with a cross-flow nebulizer and Scott spray chamber. Seeds were ground into powder, and 250 mg of dry material was digested in 20 mL of 65% HNO_3_ and 2 mL of 30% H_2_O_2_. Internal standard Yttrium (Y) was used to control digestion process quality and possible matrix effects. Measurements of mineral–nutrients were calibrated with standards for ICP from Merck. Element concentrations that exceeded the linear dynamic range were diluted using calibrated pipettes and re-analyzed.

### 2.4. In-Vitro Digestion Analysis

Seeds (5 g) from each parental line (S-91 and S-297) were crushed using a mortar and pistil followed by homogenization with 10 mL dH_2_O, with four replicates. The obtained sesame seed paste was used for mineral–nutrients bio-accessibility evaluation. An in vitro gastro-intestinal digestion model was employed with some modifications [20]. The concentration of Zn, Fe, Ca, Mg and P, were analyzed using ICP in the undigested raw sample and digestion samples: pellet (solid) and supernatant (liquid) fractions of both simulated gastric fluid (SGF) and simulated intestinal fluid (SIF) phases. To test the iron bio-accessibility in other food sources, we analyzed, whole bread wheat (*Triticum aesrivum* L.) flour, cornflakes cereals (Unilever Israel), chickpea (*Cicer arietinum* L.) seeds, chicken (*Gallus gallus domesticus*) liver, corn (*Zea mays* L.) seeds, and spinach (*Spinacia oleracea* L.), as described above.

### 2.5. QTL Analysis

DNA was extracted from the young leaf tissue (~50 mg) of five weeks-old plants of both parental lines and 149 individuals of F_2_ population (S-91 × S-297) using the CTAB protocol [21]. DNA was diluted to ~100 ng/µL and used for sequencing (Illumina high-seq 2500; A&M AgriLife Research, College Station, TX, USA). The raw sequences were used for genotypic by sequencing (GBS) marker development (3030 SNP (single nucleotide polymorphism) and 16,279 INDEL (insertions and deletions)). The markers were anchored to the sesame reference genome S_indicum_v1.0 [22] to produce a physical genetic map. QTL analyses were performed by NRGene LTD (Ness Ziona, Israel) based on the phenotyping and GBS data of 149 individual F_2_ plants. QTL visualization was conducted with IGV 2.3 software [23]. Correspondence between the QTL of different traits was determined using the hypergeometric probability function according to Peleg et al. [24]:

(1) $$P=\frac{\left(\begin{array}{c} l\\ m \end{array}\right)\left(\begin{array}{c} n-l\\ s-m \end{array}\right)}{\left(\begin{array}{c} n\\ s \end{array}\right)}$$

where *n* is the number of comparable intervals; *m* is the number of ‘matches’ (QTL of two traits with >50% overlap between their confidence intervals) declared between QTL; *l* is the number of QTLs found in the larger sample and *s* is the number of QTLs found in the smaller sample.

Major genomic regions (i.e., several overlapping QTLs) were genetically characterized for candidate genes (CG) identification. Emphasis was given to QTL associated with seed Zn and Fe concentrations. The genomic regions aligned to the sesame reference genome (S_indicum_v1.0) and genes within the interval were screened using NCBI Genome browser. The major genomic regions were re-sequenced to validate polymorphism between the two parental lines as well.

### 2.6. Statistical Analyses of Phenotypic Data

The JMP^®^ Pro ver. 15 statistical package (SAS Institute, Cary, NC, USA) was used for all statistical analyses. Bartlett’s test was used to examine the homogeneity of variance among treatments. All phenotypic variables were tested for normal distribution. Differences between sesame genotypes in quantitative traits were tested using Tukey-HSD. Pearson’s correlation coefficients were used to assess associations between the measured phenotypic traits. Comparison between the two parental lines was analyzed using Student’s *t*-test.

## 3. Results

### 3.1. Wide Phenotypic Variation in Morphological and Seed Mineral-Nutrient Concentrations

**Figure 1:**
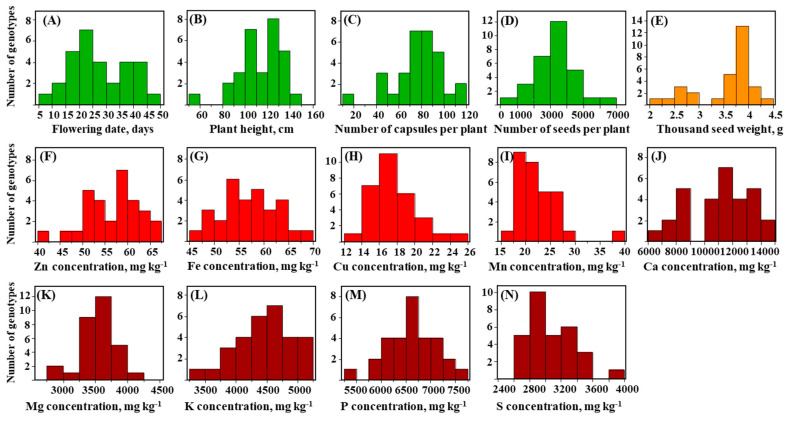
Frequency distribution of plant *phenological and morphological traits* (green): (**A**) flowering date, (**B**) plant height, (**C**) number of capsules per plant, (**D**) number of seeds per capsule, *Seed quality trait* (orange): (**E**) thousand seed weight and seed concentration of *micronutrients* (red): (**F**) zinc (**G**) iron, (**H**) copper, (**I**) manganese, and *macronutrients* (burgundy), (**J**) calcium, (**K**) magnesium, (**L**) potassium, (**M**) phosphorus, and (**N**) sulfur, in core-collection of 30 sesame genotypes.

The genetic diversity for plant morphology and seed mineral-nutrient concentrations was studied across a core-collection of 30 sesame genotypes from various geographical origins (Table S1). In accordance of being the center of diversity for sesame, the core-collection represented mostly Asia and Africa (25 genotypes). Analysis of variance (ANOVA) showed that most traits were not associated with their geographical origin (Table S2). Frequency distributions indicated normal distribution for the majority of traits, excluding plant height (PH), thousand seed weight (TSW), seed manganese (Mn) and sulfur (S) concentration (Figure 1). Flowering date (FD) displayed the highest variation (CV = 37%), ranging between 9.7 (S-49) and 45.3 (S-93) d after planting (DAP) and followed by the yield traits, seed per plant (SPP) (CV = 35.7%) and number of capsules per plant (NCPP) (CV = 26.5%) (Figure 1, Table S3). One way ANOVA showed a significant effect of the genotypes for all traits (Table S3).

Correlations analysis was used to evaluate the relationships between yield components and seed mineral–nutrients traits among the core-collection. NCPP and SPP were positively correlated (*r* = 0.90, *p* < 0.0001) and both were negatively correlated with TSW (*r* = −0.50, *p* = 0.005 and *r* = −0.73, *p* < 0.0001, respectively) (Table S4). In accordance with their geographical origin, FD and PH were positively correlated (*r* = 0.59, *p* = 0.001). PH was positively correlated with NCPP and SPP (*r* = 0.59, *p* = 0.001 and *r* = 0.69, *p* < 0.0001, respectively). Seed Fe concentration was positively correlated with most bivalent cations Zn, Cu and Mg. Seed P was positively correlated with Zn, Fe, Cu, Mg and S and showed no correlation with Mn, Ca or K (Table S4). S-91 exhibited high seed Zn (66.08 mg/kg) and Fe (66.94 mg/kg) concentrations, with relatively moderate P concentration (6673.75 mg/kg), hence, it was selected for the further genetic dissection of seed mineral–nutrients (Table S3).

### 3.2. S-91 × S-297 Population Exhibited Range of Interrelationships between Plant Morphological and Seed Quality Traits

The two parental lines exhibited significant differences in morphological and quality traits (Table 1, Figure S1). S-91 had an indehiscent capsule morphology, whereas S-297 had a dehiscent capsule. S-91 also showed significantly longer capsules and a higher TSW compared with S-297. S-297 exhibited earlier flowering (18 ± 1.05 vs. 25 ± 1.36), at a lower node (3 ± 0.20 vs. 5 ± 0.71), with more nodes per plants as compared with S-91. A significantly higher seed Zn concentration (87.5 ± 5.5 vs. 58.4 ± 1.7 mg/kg), Fe (97.3 ± 11.2 vs. 76.4 ± 6.6 mg/kg) and Cu (23.2 ± 1.0 vs. 14.5 ± 1.0 mg/kg) were found in S-91 when compared with S-297 (Table 1, Figure S1).

**Figure 2:**
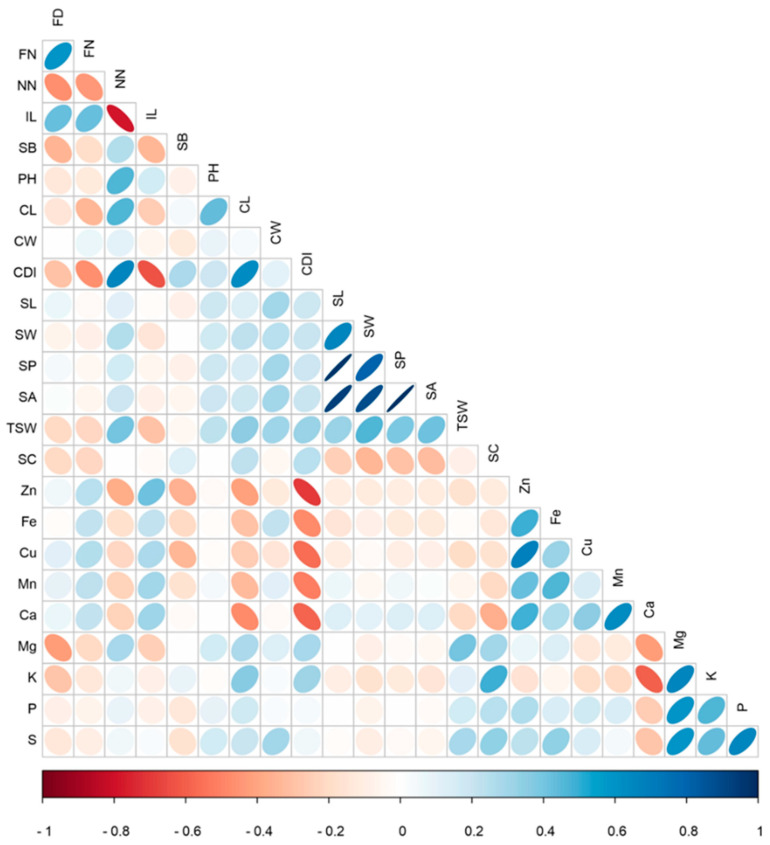
Person correlation matrix in the F_2_ population (S-91 × S-297) between the phenotypic traits: FD, flowering date; FN, First flowering node; NN, number of nodes; IL, internode length; SB, number of secondary branches; PH, plant height; CL, capsule length; CW, capsule width; CDI, capsule dehiscence index; SL, seed length; SW, seed width; SP, seed perimeter; SA, seed area; TSW, thousand seed weight; SC, seed color; Zn, zinc; Fe, iron; Cu, copper; Mn, manganese; Ca, calcium; Mg, magnesium; K, potassium; P, phosphorus; and S, sulfur. Colors indicate level of correlation (r) from positive correlation (blue) to negative (red). Circle size indicates the level of significance (Data can be found in Table S5).

Phenotypic characterization of the F_2_ population in the field revealed wide variation with transgressive segregation for most traits (Table 1 and Table S1). For example, Zn and Fe concentrations exceeded S-91 values by 19% and 16%, respectively. Normal distribution was found for most traits, except for FD, CDI, SB, Zn and Ca. FD was positively correlated with FN and IL (*r* = 0.59, *p* < 0.0001 and *r* = 0.42, *p* < 0.0001, respectively) and negatively correlated with NN and TSW (*r* = −0.46, *p* < 0.0001 and *r* = −0.21, *p* = 0.014, respectively). CW was found to positively correlate with all seed morphological traits (i.e., SL, SW, SP, SA and TSW). CL was positively correlated with SW, SA and TSW. CW and CL did not correlate significantly with each other (Figure 2, Table S5). CDI was found to significantly correlate with all traits except for CW, P and S. SC was negatively correlated with FD and the seed morphological traits SL, SW, SP and SA. TSW was positively correlated with Mg, P and S and negatively correlated with Zn, Cu and Ca. NN was negatively correlated with all micronutrients (i.e., Zn, Fe, Cu and Mn), while IL exhibited the opposite trend and correlated positively with all micronutrients. All micronutrients were positively correlated with each other and so were the macronutrients, except for Ca, which correlated negatively with the other macronutrients tested and positively with all micronutrients. Ca was also found to correlate negatively with TSW and seed color. Seed P concentration was significantly correlated with the micronutrients Zn and Cu and did not correlate with Fe and Mn. Significant positive correlations were found between Fe and the bivalent cations Zn, Cu, Mn and Ca (Figure 2, Table S5).

### 3.3. Constructing an High-Density Genetic Map and QTL Analysis

**Figure 3:**
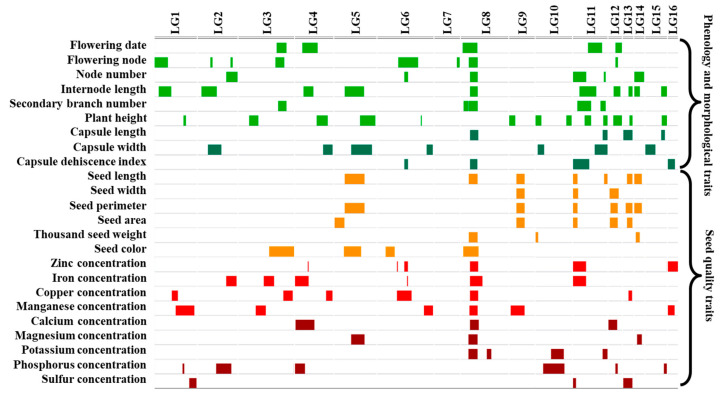
Genomic architecture of agronomical important traits in sesame. For each trait, the significant associated loci indicated by rectangles across sesame genome linkage groups (LG). Plant (green) and capsule (dark green) phenological and morphological traits. Seed quality (orange), micronutrients (red) and macronutrients (burgundy) concentrations.

A high-density genetic linkage map consisting of 19,309 (3030 SNPs and 16,279 INDELs) markers assigned to 16 linkage groups (LG), was constructed. A bin-map with 2339 bin markers accounting for a total length of 1497 cM was created following recombination events across the population and the combination of adjacent SNPs, possessing the same genotype in an interval, into bins. LG sizes ranged from 17.9 to 177.2 cM, with an average of 0.9 cM between adjacent bins (Table S6). The QTL analysis using the phenotypic data collected for 24 traits and the bin-map molecular marker data revealed 134 significant QTL across all 16 sesame LGs (Table 2 and Table S7, Figure 3). In general, each parent contributed 50% of the favorable alleles (67 and 66 for S-91 and S-297, respectively).

### 3.4. QTL for Phenology and Morphological Traits

Detailed biometric parameters of QTL detected for each trait are the following:

*Flowering date*: A total of six significant QTL were associated with FD with LOD (logarithm of odds) scores ranging between 2.1 and 4.9 and explaining 6.3–14% of the variance (Table 2). The S-297 allele conferred higher FD in all loci (LG3, 4, 8, 11, 11, 12) (Table S7).

*Plant morphology traits*: Flowering node (FN), node number (NN), internode length (IL) and number of secondary branches (SB) conferred by 8, 6, 10, and 5 QTL, respectively, with most QTL contributed by S-297 (25 loci) (Table 2 and Table S7).

*Plant height*: A total of 13 significant QTL were associated with PH with LOD scores range between 2.0 and 4.7 and explain 5.3–11.9% of the variance (Table 2). Higher PH was conferred by the S-91 allele at ten loci (LG1, 4, 5, 9, 10, 11, 11, 12, 13, 15) and by the S-297 allele at three loci (LG3, 6, 10) (Table S7).

*Capsule length*: A total of four significant QTL were associated with CL with LOD scores ranging between 2.4 and 19.7, explaining 7.1–45.4% of the variance (Table 2). Longer CL was conferred by the S-91 allele at three loci (LG11, 13, 15) and by the S-297 allele at LG 8 (Table S7).

*Capsule width*: A total of seven significant QTL were associated with CL with LOD scores ranging between 2.1 and 4.0, explaining 6.2–11.5% of the variance (Table 2). Longer CL was conferred by the S-91 allele at three loci (LG4, 6, 10, 15) and by the S-297 allele at three loci (LG2, 5, 11) (Table S7).

*Capsule dehiscence index*: A total of four significant QTL were associated with CDI with LOD scores ranging between 2.7 and 95.1, explaining 6.3–76.8% of the variance (Table 2). S-91 allele conferred indehiscence capsule phenotype (lower CDI) for all four loci (LG6, 8, 11, 16) (Table S7). 

*Seed morphology traits*: seed length (SL), Seed width (SW), Seed perimeter (SP) and Seed area (SA) conferred by 7, 3, 6, and 5 QTL respectively, with most QTL contributed by S-297 (13 loci) (Table 2 and Table S7).

### 3.5. QTL for Seed Quality Traits

*Thousand seed weight*: A total of three significant QTL were associated with TSW with LOD scores ranging between 2.6 and 4.8, explaining 7.7–13.6% of the variance (Table 2). Higher TSW was conferred by the S-91 allele at two loci (LG10, 14) and by the S-297 allele at LG 8 (Table S7). 

*Seed color*: A total of four significant QTL were associated with SC with LOD scores ranging between 2.1 and 12.1, explaining 6.3–31.0% of the variance (Table 2). The S-297 allele conferred higher SC for all loci (LG3, 5, 6, 8) (Table S7).

*Seed zinc concentration*. A total of six significant QTL were associated with Zn with LOD scores ranging between 2.4 and 19.9, explaining 7.2–45.7% of the variance (Table 2). Higher Zn was conferred by the S-91 allele at five loci (LG 4, 6, 8, 11, 16) and by the S-297 allele at one locus (LG6).

*Seed iron concentration*. A total of six significant QTL were associated with Fe with LOD scores ranging between 2.1 and 7.9, explaining 6.1–21.6% of the variance (Table 2). Higher Fe was conferred by the S-91 allele at five loci (LG2, 3, 6, 8, 11) and by the S-297 allele at one locus (LG4) (Table S7).

*Seed copper concentration*. A total of six significant QTL were associated with Cu with LOD scores ranging between 2.1 and 11.8, explaining 6.3–30.4% of the variance (Table 2). Higher Cu was conferred by the S-91 allele at all six loci (LG1, 3, 4, 6, 8, 13) (Table S7).

*Seed manganese concentration*. A total of six significant QTL were associated with Mn with LOD scores ranging between 2.4 and 8.2, explaining 7.1–22.2% of the variance (Table 2). Higher Mn was conferred by the S-91 allele at four loci (LG1, 6, 8, 16) and by the S-297 allele at one locus (LG 3). For one locus, qMn-5 (LG9), we could not separate the contribution of the parental lines (Table S7).

*Seed calcium concentration*. A total of three significant QTL were associated with Ca with LOD scores ranging between 2.1 and 7.8, explaining 6.1–21.4% of the variance (Table 2). Higher Ca was conferred by the S-91 allele at two loci (LG 8, 12) and by the S-297 allele at one locus (LG4).

*Seed magnesium concentration*. A total of three significant QTL were associated with Mg with LOD scores ranging between 2.3 and 4.2, explaining 6.8–12% of the variance (Table 2). Higher Mg was conferred by the S-91 allele at one locus (LG14) and by the S-297 allele at two loci (LG5, 8) (Table S7).

*Seed potassium concentration*. A total of four significant QTL were associated with K with LOD scores ranging between 2.6 and 3.7, explaining 7.5–10.7% of the variance (Table 2). Each parental line contributed the allele for higher K at two of the loci, LG 10 and 11 (S-91) and two loci on LG8 (S-297).

*Seed phosphorus concentration*. A total of six significant QTL were associated with P with LOD scores ranging between 2 and 2.7, explaining 5.9–7.8% of the variance (Table 2). Higher P was conferred by the S-91 allele at five loci (LG1, 2, 4, 10, 12) and by the S-297 allele at one locus (LG 15) (Table S7).

*Seed sulfur concentration*. A total of three significant QTL were associated with S with LOD scores ranging between 2.5 and 4.3, explaining 7.2–12.5% of the variance (Table 2). Higher S was conferred by the S-91 allele at two loci (LG 11, 13) and by the S-297 allele at one locus (LG 1) (Table S7).

### 3.6. Candidate Genes Associated with Seed Mineral-Nutrient Concentration

In order to scan for putative candidate genes (CG), we focused on six hotspots containing overlapping QTL for several traits on LG6 (13653153–13744314), LG8 (4550182–7125811), LG11 (310665–1216709, 5566003–5767772 and 14205927–14426041), and LG16 (14816–3048510), harboring 1, 53, 89, 11, 38 and 189 genes, respectively (Table S8). Out of these 381 potential CG, 285 were annotated into diverse functions, while the other 96 were uncharacterized, with an unknown function. Focusing on CG associated with seed nutrients and morphological traits, we selected 36 potential CG that directly matched the established QTL effects (Table 3). Comparison between parental lines sequences revealed 13 CG that exhibited substantial polymorphism that is likely to affect their function, while in the rest 23 CG no sequence polymorphism was found (Table 3 and Table S9). For example, QTL qK-1 conferring seed potassium concentration (LG8) included, SKOR-like and SKOR potassium channels (LOC105167760 and LOC105167785, respectively). SKOR is a K^+^ selective outward rectifying potassium channel expressing in the root tissue and has an important role in K^+^ translocation from root to shoot via the xylem [25]. A substantial deletion of 545 bp was detected in LOC105167785 of S-91. QTL qZn-5 and qFe-6 affecting seed zinc and iron concentrations (LG11) include LOC105173373 (phosphate starvation response 1-like protein; PHR1). Its homolog gene AtPHR1 in Arabidopsis was found to be involved in regulation of iron, zinc phosphate and sulfate homeostasis [26]. Additionally, two ferric reduction oxidases (FRO) 2 and 2-like (LOC105173155 and LOC105173156, respectively) were also identified within this QTL. FRO2 known to reduce Fe^3+^ to Fe^2+^ which is an essential step for Fe uptake from the soil. Overexpression of FRO2 homolog in Arabidopsis conferred tolerance to iron deficiency [27]. Three genes encoding to a cyclic nucleotide-gated ion channel 1-like (CNGC1) (LOC105173138, LOC105173087 and LOC105173088) were included in qZn-5, qFe-6 and qS-2 (LG11). A missense mutation was detected at 1516 bp position of LOC105173138 mRNA, which led to an amino acid substitution from Val506 in S-297 to Leu506 in S-91. The homolog AtCNGC1 is primarily expressed in Arabidopsis roots, and CNGC mutants were found to be related to metal ion (i.e., K^+^, Na^+^, Ca^2+^ Pb^2+^ and Ni^2+^) homeostasis, uptake, and transport [28]. QTL qZn-6 conferring seed zinc concentration (LG16) includes zinc transporter 8 (LOC105178590) and zinc transporter 8-like (LOC105178589) genes that in rice was shown to affect zinc concentration [29]. A SNP at 29 bp position of the gene transcript within the first exon of LOC105178590, resulted in an amino acid substitution of Gly10 in S-91 to Ala10 in S-297. LOC105178589, consisted two SNP at position 154 bp and 279 bp of the gene transcript, which resulted in amino acid substitutions from Ser52 and Phe102 in S-91 to Gly52 and Ser102 in S-297, respectively.

### 3.7. Bio-Accessibility of Seed Mineral–Nutrients

To study the connection between high mineral-nutrient concentration and their bio-accessibility level, we conducted an in vitro bio-accessibility experiment [20]. We compared sesame seed paste iron bio-accessibility and various plant- and animal-based food sources (whole wheat grains, corn grains, cornflakes, chickpea, spinach and chicken liver) (Table S10). Examination of the simulated gastric fluids (SGF) showed that within the solid fraction, chicken liver exhibited the highest iron concentration (63.53 mg/kg), while among the plant-based sources, iron concentration of sesame paste was the highest (33.78 mg/kg) and corn was the lowest (3.56 mg/kg). Within the SGF liquid fraction, spinach had the highest iron concentrations (2.03 mg/kg), sesame paste was second (1.94 mg/kg) and chicken liver was the lowest (0.16 mg/kg). Examination of the simulated intestinal fluids (SIF) showed that within the solid fraction, cornflakes had the highest iron concentration (105.65 mg/kg), while sesame paste had much lower concentration (16.55 mg/kg) and chicken liver had the lowest concentration (1.60 mg/kg). Within the SIF liquid fraction, chicken liver exhibited again the highest iron concentration (3.18 mg/kg), sesame paste iron concentration was the highest among the plant-based sources (2.67 mg/kg) and corn was the lowest (0.34 mg/kg).

**Figure 4:**
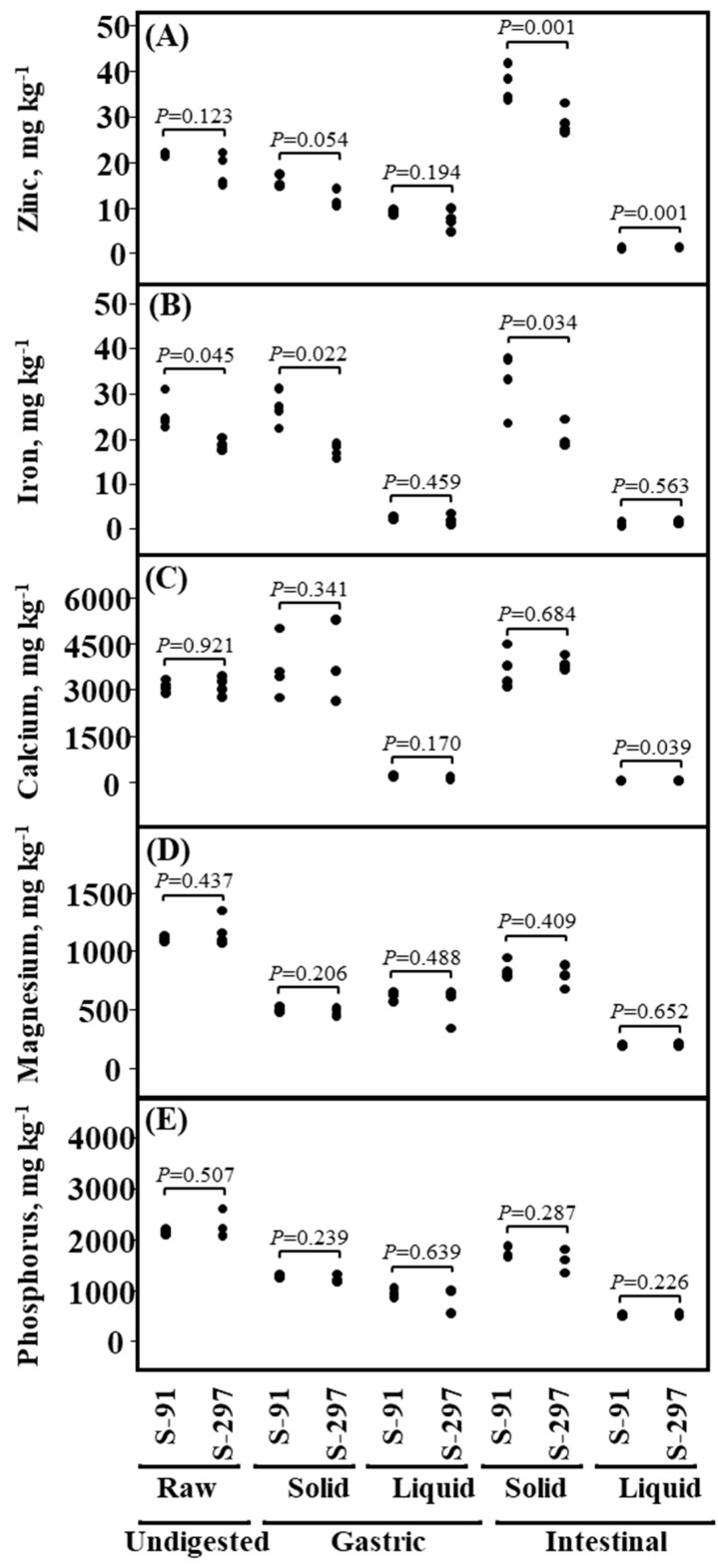
Bio-accessibility of mineral–nutrients between S-91 and S-297 lines. In vitro digestion of seed paste for (**A**) zinc, (**B**) iron, (**C**) calcium, (**D**) magnesium and (**E**) phosphorus concentrations in the different phases of the digest (Undigested, Gastric and Intestinal) in each fraction (Raw sample, Solid and Liquid). *p*-values indicate differences between the two genotypes (S-91 and S-297) as determined by *t*-test (*n* = 4).

In order to test whether sesame seeds originated from different sesame lines will differ in their mineral–nutrients bio-accessibility, we conducted a comprehensive in vitro digestion analysis of both parental lines (S-297 and S-91) and assessed the bio-accessibility of five important mineral–nutrients (i.e., Zn, Fe, Ca, Mg and P) (Figure 4, Table S11). The initial concentration of all mineral–nutrients in the undigested raw sesame paste of both parental lines showed no significant differences, except for Fe (25.5 ± 1.9 vs. 18.5 ± 0.7 mg/kg for S-91 and S-297, respectively). In general, for both gastric and intestinal phases, the solid fractions contained higher mineral–nutrients concentrations as compared to the liquid fractions. Liquid intestinal (SIF supernatant; indicator of micronutrients bio-accessibility) differ significantly in Zn concentration between S-91 and S-297 (1.2 ± 0.1 vs. 1.4 ± 0.1 mg/kg, respectively) and Ca concentration (71.7 ± 3.4 vs. 58.7 ± 5.3 mg/kg) (Figure 4, Table S11).

## 4. Discussion

Mineral-nutrient malnutrition is a global health problem [30], as consequence of cereal-based diets, which fail to provide sufficient amounts of mineral–nutrients. While plant breeding programs focus on increasing yield, an equally important quest that remains a principal concern but is largely overlooked in breeding programs is the nutritional value of food crops. Plants biofortification is the most promising, cost-efficient, strategy to reduce malnutrition. Here we used the wide natural genetic diversity in sesame, to study the seed mineral-nutrient concentration and dissect its genetic architecture. Our results demonstrate the nutritional potential of the sesame genepool as a source of novel alleles to enhance the nutritional quality and set the basis for future sesame breeding programs.

Sesame is an ‘orphan crop’ that mainly grown using traditional practices, and as a consequence, it possesses wide allelic repertoire for desirable traits. The core-collection exhibited wide genetic diversity for morpho-phenological traits (Figure 1). Similarly, wide diversity was found among sesame collections for various traits, such as flowering time, plant height, branches number and seed yield per plant [31,32,33]. TSW and PH showed negative correlation, which may indicate a tradeoff between the sources invested in vegetative growth vs. seed filling, as observed in rapeseed [34]. The strong positive correlations between PH and the traits FD, NCPP and SPP suggest that the transition to the reproductive stage (i.e., induction of flowering), reduces vegetative growth, and as a consequence restrict plant height [35]. The highly significant positive phenotypic correlation between NCPP and SPP (*r* = 0.90, *p* < 0.0001) and the negative correlation between those traits and TSW (Table S4), may indicate compensation between yield components via morphological modifications [7].

Mineral-nutrient diversity of most staple crops was shown to have narrow diversity, due to the genetic bottleneck associated with domestication and breeding (i.e., wheat [36], maize [37] and rice [38]). On the other hand, alleles originated in landraces and wild progenitors offer ample diversity for seed nutrients, as was found for sesame in the current study (Figure 1), barley landraces [39], wild wheat [40] and wild rice [41]. Zn and Fe exhibited a highly significant positive phenotypic correlation (*r* = 0.65, *p* < 0.0001) (Figure 2, Table S5). Similarly, Zn and Fe were positively associated in wild and domesticated wheat [40] and bean [42], which suggest common molecular mechanisms controlling these minerals uptake and metabolism. Fe and Zn are regulated and distributed by specific mechanisms, yet, similar chemical properties enable these two divalent cations undergo through mutual regulation mechanisms. Moreover, the metal-transporters involved in Fe or Zn uptake, also mediate transport of other divalent cations [43]. For example, in *Arabidopsis*, the iron-regulated transporter 1 (IRT1, a member of the ZIP family of metal transporters), was also found to uptake Zn and Mn [44].

In plants, up to 85% of the phosphorus (P) is accumulated in the seeds in the form of phytate, therefore P can be used as an indicator for phytate content [45,46]. Phytate is known to chelate metal ions, especially Zn but also Fe, Ca, K, Mn and Mg, making them insoluble, thus, inhibiting their digestion and absorption in the gastrointestinal tract [47,48]. A strong positive correlation between P and the cations Zn, Fe and Mg were found in the present study (Figure 2), as well as for other crop-plants such as wheat [36], maize [37] and bean [48].

While sesame is known as a highly nutritional food-source, genetic studies aim to uncover the genetic architecture of seed nutrient concentration are very scarce. In general, our genetic dissection of yield components and seed quality traits revealed numerous genomic regions and co-localizations of different QTL affecting different traits across sesame genome. A strong phenotypic (*r* = −0.7, *p* < 0.0001) and genotypic (*p* < 0.001) correlation was found between seed Zn concentration and CDI on LG6, LG8, LG11 and LG16 (Figure 2; Figure 3, Table S5), which was also supported by traits phenotypic correlation. In three of these loci a significant correlation was also found with seed Fe concentration and NN, which was supported by significant phenotypic correlation as well. Interestingly, in all four loci, S-91 was the favorable allele (higher nutrient concentration), with exception of NN which was contributed by S-297 (Table S7). The strong correlation between qCDI-2 QTL and all seed micronutrients may suggest that in this locus there is a major regulatory CG affecting both plant development and nutrients homeostasis.

QTL conferring Zn and Fe micro-nutrients were found to co-localize at four different LG (4, 6, 8 and 11) across the genome. Similar compatibility between Zn and Fe phenotypic and genotypic correlations was also reported for rice [49] and tetraploid wheat [24]. These findings suggest a strong genetic association between mechanisms affecting seed Zn and Fe concentrations. In order to maintain plant metal homeostasis, a complex network of metal uptake, trafficking, transportation, accumulation and sequestration mechanisms is essential. These processes are tightly regulated by several genes, which are not necessarily selective to a specific metal.

Prominently, 16 QTLs were co-localized on LG8, to a narrow confidence interval of 2,575,630 bp with major QTL qCDI-2 (Figure 3, Table S7), including seven seed mineral–nutrients concentration traits (i.e., Zn, Fe, Cu, Mn, Ca, Mg and K), five morpho-phenological traits (FD, FN, NN, IL and SB), capsule morphology traits (CL) and three seed morphology traits (SL, TSW and SC). Interestingly, higher mineral–nutrients concentration was conferred by the S-91 alleles in most QTL, except qMg-2 and qK-2. On the other hand, desirable characters of morpho-phenological traits were contributed by the S-297 alleles in most traits (Table S7; Figure 3). Recently, the SiCL1 gene controlling leaf curling was mapped to a similar location [50]. Hence, it is yet to be determined whether this genomic region contains a cluster of several tightly linked genes or one pleotropic gene affecting various developmental processes in sesame.

The identification of CG that may affect seed mineral–nutrients and morphological traits can serve as a solid basis for future studies combining transcriptional expression, allele mining and eventually fine mapping of promising CG. While QTL analysis cannot provide resolution at a single gene level, we focused on several hotspots with overlapping QTL and narrowed down the interval for high confidentiality. Despite the relatively large number of genes that reside within the six target QTL intervals, selection according to annotation enabled us to focus on potential CG (Table 3). Further selection upon CG polymorphism data indicated several CG with a high potential to affect mineral–nutrients homeostasis both directly or indirectly (Table 3 and Table S9). Genes encoding metal ion transporters can directly contribute to the increased mineral-nutrient concentration in the seeds. The Shaker family is a major voltage dependent K^+^ channel protein family. One key family member is SKOR, a selective potassium channel related to K metabolism. Within the genomic region of the major QTL, qK-1 affects seed potassium concentration (explaining 10.7% of the variance), and two SKOR-like and SKOR genes (LOC105167760 and LOC105167785, respectively) were found. The 545 bp deletion found in S-91 SiSKOR (LOC105167785), eliminating a part of the 7th intron and almost the entire 8th exon, which impairs the protein’s conserved cyclic nucleotide binding domain (cNBD). This domain is thought to mediate the interactions between the channel tetramer subunits [25]. In *Arabidopsis*, a mutation in the fourth exon of *AtSKOR1* resulted in a knockout mutant exhibited 50% decrease in shoot K^+^ content [51]. Notably, higher seed potassium concentration values were contributed by the S-297 allele at qK-1 locus.

Plant cyclic nucleotide-gated ion channels (CNGC) comprise a plasma membrane non-selective cation conducting channels, containing a cNBD domain, which, in contrast to SKOR channels, opens the channel gate upon cyclic nucleotide monophosphate (cNMP) ligand binding [52]. The polymorphism detected in LOC105173138, one of the three SiCNGC1 genes found within the mutual confidence interval of qZn-5, qFe-6 and qS-2, led to an amino acid substitution at the protein 506 position. The 506 position in SiCNGC1 is corresponded to a highly conserved Leu residue found in the phosphate binding cassette region of the cNBD [52]. Interestingly, S-91 possessed the conserved Leu506 while S-297 acquired Val506. Hence, the favorable allele associated with higher seed Zn, Fe and S concentrations was contributed by S-91.

The ZIP family protein is a large family of zinc transporters that involved in Zn^2+^ and other divalent metal cations (Fe^2+^, Mn^2+^, Cu^2+^ and Cd^2+^) transport [53]. Two zinc transporters, 8 (LOC105178590) and 8-like (LOC105178589), were co-localized with qZn-6 and qMn-6 on LG16 (Table 3 and Table S7). Previously, it was suggested that the residue at position 102 in the second transmembrane domain (TM2) is a conserved hydrophobic residue. The hydrophobic nature is believed to be involved in the blocking of metals at the extracellular surface [44]. In comparison to S-297 Ser102, S-91 consists a more hydrophobic residue of Phe102. Interestingly, the favorable allele for qZn-6 and qMn-6 QTL on LG16, was donated by S-91.

Genes encoding proteins associated with metal ion chelation can have an indirect effect on minerals homeostasis. For example, proteins related to organic acids (e.g., phytate, citrate and ascorbate) metabolism and plant redox known to affect metal ion homeostasis, such as inositol hexakisphosphate and diphosphoinositol-pentakisphosphate kinase 2-like (LOC105167762), isocitrate dehydrogenase (LOC105167815), ferric reduction oxidases 2 and 2-like (LOC105173155 and LOC105173156, respectively), ascorbate transporter (LOC105173161), citrate synthase (LOC105173380), nicotianamine aminotransferase A (LOC105178476), isocitrate lyase (LOC105178507) and aconitate hydratase (LOC105178516).

An important, yet poorly investigated aspect is the impact of high seed nutrient concentration on the bio-accessibility level, which can also be affected from the level of anti-nutritional factors. Chelation of mineral-nutrient cations by phytate and competition between bivalent cations such as Zn^2+^, Mn^2+^, Ca^2+^ and Mg^2+^ with Fe^2+^ is known to impair the absorbance of the latest [54,55,56,57]. Here we applied an in vitro digestion approach simulating the bio-accessibility of key micro-nutrients in an attempt to test the connection between the identified QTL and bio-accessibility. Previous studies suggested that the major part of mineral–nutrients are being absorbed in the liquid fraction of the small intestines, that is, SIF liquid (supernatant) is the most bio-accessible fraction for micronutrients [58]. To evaluate the potential of sesame seeds to provide good source of iron, we tested the iron bio-accessibility of various food sources including, chicken liver, whole wheat grains, corn, cornflakes, chickpea and spinach (Table S10). Among all plant-based sources, sesame seed paste exhibited the highest iron liquid fraction SIF values (2.67 mg/kg compared with average of 0.77 mg/kg), which was only second to chicken liver (3.18 mg/kg).

A comprehensive in vitro digestion analysis of the mineral–nutrients’ bio-accessibility of the two parental lines (S-297 and S-91) showed that the highest mineral–nutrients concentrations were found in the solid portion of the digest in both gastric and intestinal phases (Figure 4, Table S11), suggesting that it is less bio-accessible (which in turn can imply on their bio-availability as well). Notably, although S-297 had lower levels of zinc in the raw sesame paste, its zinc bio-accessibility seemed to be higher. In terms of iron, the in vitro model clearly demonstrates that the differences in seed iron concentration between the two parental lines also expressed in the liquid and solid parts of the digest, in both gastric and intestinal phases. Our results suggest that while pyramiding of QTL for nutrients concentration could be an efficient strategy to improve nutritional content in plant-based food, the nutrients are not always available. Thus, further research is needed to identify major genes that regulate nutrient bio-accessibility.

## 5. Conclusions

Breeding food crops with enhance nutrient concentration in the seeds is a low-cost, sustainable strategy to alleviate malnutrition. Increasing seed mineral–nutrients concentrations are also likely to improve seed germination and establishment under changing climates. The sesame genepool offers abundant genetic diversity for seed minerals. To the best of our knowledge, this is the first report on QTL for mineral–nutrients in sesame. The identified associations between QTL affecting different mineral–nutrients suggest physiological coupling of certain processes that govern mineral-nutrient accumulation in sesame. Few genomic regions (LG6, 8, 11, 16; Table S8) were found to harbor QTL clusters for several minerals. These regions offer unique opportunities for synchronous improvement of Zn, Fe and other mineral–nutrients in sesame seeds. Nevertheless, genomic regions associated with only one or few minerals should not be overlooked as they may confer other, mineral-specific, mechanisms. The concurrent mapping of QTL for several minerals as well as the dissection of their inter- and intra-relationships provide an insight into the functional basis of the physiology, genomic architecture and evolution of mineral-nutrient accumulation in sesame and other crops.

## Figures and Tables

**Table 1:** Mean values and ranges of plant morphological traits and seed mineral-nutrient concentration of 149 F_2_ lines (S-91 × S-297) as well as the two parental lines.

Trait	Code	S-91	S-297	F_2_ Population	
				Mean	Range
**Flowering date (days)**	FD	25 ± 0.14	18 ± 0.11	17.8	14–29
**Flowering Node**	FN	5 ± 0.7	3 ± 0.2	3.4	2–5
**Node Number**	NN	29 ± 1.9	36 ± 1.3	34.1	20–43
**Internode Length (cm)**	IL	4.9 ± 0.1	4.1 ± 0.2	4.1	2.7–6.5
**Number of Secondary Branches**	SB	1 ± 0.5	0 ± 0.2	0.7	0–5
**Plant Height (cm)**	PH	142 ± 11.7	147 ± 8.3	134.2	100–170
**Capsule Length (mm)**	CL	42.9 ± 1.3	28.8 ± 0.7	34.5	20.6–48.0
**Capsule Width (mm)**	CW	6.46 ± 0.22	5.71 ± 0.1	6.34	5.22–8.43
**Capsule Dehiscence Index**	CDI	1	5	4.2	1–5
**Seed Length (mm)**	SL	3.31 ± 0.02	3.41 ± 0.03	3.50	3.08–3.86
**Seed Width (mm)**	SW	2.1 ± 0.0	2.1 ± 0.0	2.1	1.9–2.3
**Seed Perimeter (mm)**	SP	10.6±0.1	10.9 ± 0.1	11.21	10.1–12.3
**Seed Area (mm^2^)**	SA	5.32 ± 0.11	5.56 ± 0.10	5.80	4.75–6.90
**Thousand Seed Weight (g)**	TSW	3.35 ± 0.14	2.97 ± 0.07	3.11	0.97–3.99
**Seed Color (RGB index)**	SC	153.1 ± 2.3	159.1 ± 2.5	155.3	128.1–198.3
**Zinc (mg/kg)**	Zn	87.5 ± 5.5	58.4 ± 1.7	71.1	45.5–108.6
**Iron (mg/kg)**	Fe	97.3 ± 11.22	76.4 ± 6.6	82.9	53.8–116.0
**Copper (mg/kg)**	Cu	23.2 ± 1.1	14.5 ± 1.0	17.2	11.5–23.5
**Manganese (mg/kg)**	Mn	19.3 ± 0.5	17.8 ± 1.6	16.0	11.8–22.2
**Calcium (mg/kg)**	Ca	12,591.2 ± 397.9	11,334.6 ± 947.9	10,693.6	4171.2–19,857.3
**Magnesium (mg/kg)**	Mg	4109.7 ± 122.2	4357.9 ± 90.8	4232.9	3433.0–4749.7
**Potassium (mg/kg)**	K	5577.2 ± 149.9	5519.8 ± 214.9	5434.4	4061.4–7201.0
**Phosphorus (mg/kg)**	P	8574.9 ± 239.4	9165.3 ± 81.1	8945.3	7777.9–9895.5
**Sulfur (mg/kg)**	S	3635.7 ± 64.1	3607.9 ± 183.2	3703.5	3082.6–4248.0

**Table 2:** Summary of QTL detected in F_2_ population (S-91 × S-297) associated with plant phenology and morphology, capsule morphology, seed morphology and seed quality traits.

Trait	#QTL	LOD ^a^	PVE (%) ^b^	Favorable Allele ^c^	
				S-91	S-297
**Plant Phenology and Morphology**					
Flowering date	6	2.1–4.92	6.3–14	0	6
Flowering node	8	2.56–7.57	7.6–20.7	2	6
Node number	6	2.59–19.46	7.7–45	1	5
Internode length	10	2–14.93	5.0–31.2	1	9
Number of secondary branches	5	3.03–4	8.9–11.6	0	5
Plant height	13	2–4.66	5.3–11.9	10	3
**Capsule Morphology**					
Capsule length	4	2.39–19.73	7.1–45.4	3	1
Capsule width	7	2.1–3.99	6.2–11.5	4	3
Capsule dehiscence index	4	2.66–95.1	6.3–76.8	4	0
**Seed Morphology**					
Seed length	7	2–6.4	5.9–17.8	2	5
Seed width	3	2.81–5.11	8.3–14.5	1	2
Seed perimeter	6	2.45–6.35	7.2–17.7	3	3
Seed area	5	2.3–5.77	6.8–16.2	2	3
**Seed Quality**					
Thousand seed weight	3	2.63–4.77	7.7–13.6	2	1
Seed color	4	2.14–12.09	6.3–31	0	4
Seed Zn concentration	6	2.43–19.92	7.2–45.7	5	1
Seed Fe concentration	6	2.05–7.94	6.1–21.6	5	1
Seed Cu concentration	6	2.13–11.8	6.3–30.4	6	0
Seed Mn concentration	6	2.4–8.17	7.1–22.2	4	1
Seed Ca concentration	3	2.05–7.84	6.1–21.4	2	1
Seed Mg concentration	3	2.28–4.16	6.8–12	1	2
Seed K concentration	4	2.55–3.7	7.5–10.7	2	2
Seed P concentration	6	2–2.65	5.9–7.8	5	1
Seed S concentration	3	2.45–4.34	7.2–12.5	2	1
**Total**	134			67	66

^a^ LOD (logarithm of odds) scores that were found significant when comparing hypotheses H_1_ (There is a QTL in the chromosome) versus H_0_ (There is no effect of the chromosome on the trait), using 1000 permutation test; ^b^ Proportion of phenotypic variation explained by the QTL; ^c^ The determination of favorable alleles contributing to a specific trait was based on the following: lower values of flowering date (i.e., earliness) and higher values of the other phenology and morphology traits, higher values of capsule length and width, lower value of capsule dehiscence index, higher values of seed morphology, and higher values of seed mineral-nutrient concentrations.

**Table 3:** A list of candidate genes residing within the hotspot of multi-traits QTL intervals.

LG	Interval	Trait	CG ID	Annotated Function
**LG8**	4550182–7125811	FD, FN, NN, IL, SB, CL, CDI, SL, TSW, SC, Zn, Fe, Cu, Mn, Ca, Mg, K	LOC105167760	Potassium channel SKOR-like
			LOC105167785	Potassium channel SKOR
			LOC105167762	Inositol hexakisphosphate and diphosphoinositol-pentakisphosphate kinase 2-like
			LOC105167788	Ethylene-responsive transcription factor 1B-like
			LOC105167789	Ethylene-responsive transcription factor 1B-like
			LOC105167791	Ethylene-responsive transcription factor 1B-like
			LOC105167765	Transcription repressor KAN1
			LOC105167815	Isocitrate dehydrogenase [NADP]
**LG11**	310665–1216709	NN, CDI, SL, SW, SP, SA, Zn, Fe, S	LOC110012885	Ethylene-responsive transcription factor ERF023-like
			LOC105173138	Cyclic nucleotide-gated ion channel 1-like
			LOC105173087	Cyclic nucleotide-gated ion channel 1-like
			LOC105173088	Cyclic nucleotide-gated ion channel 1-like
			LOC105173140	MYB-like transcription factor ETC3
			LOC105173141	Probable WRKY transcription factor 30
			LOC105173122	Heavy metal-associated isoprenylated plant protein 39-like
			LOC105173253	Probable polygalacturonase
			LOC105173155	Ferric reduction oxidase 2
			LOC105173156	Ferric reduction oxidase 2-like
			LOC105173161	Ascorbate transporter
**LG11**	5566003–5767772	NN, IL, SB, PH, CDI, Fe, Zn	LOC105173373	Protein PHOSPHATE STARVATION RESPONSE 1-like
			LOC105173380	Citrate synthase
**LG11**	14205927–14426041	NN, SB, PH, CL, CW, SL, P	LOC105174482	Transcription factor LHW
			LOC105174515	Transcription factor MYB1
**LG16**	14816-3048510	CDI, Zn, Mn	LOC105178592	Transcription factor bHLH30-like
			LOC105178450	Transcription factor TCP10-like
			LOC105178476	Nicotianamine aminotransferase A
			LOC105178598	WRKY transcription factor 6
			LOC105178495	Transcription factor MYB101
			LOC105178506	MYB-like transcription factor ETC1
			LOC105178507	Isocitrate lyase
			LOC105178516	Aconitate hydratase
			LOC105178613	Calcium-binding protein PBP1-like
			LOC105178537	Transcription factor MYB39-like
			LOC105178559	Ethylene-responsive transcription factor 4-like
			LOC105178589	Zinc transporter 8-like
			LOC105178590	Zinc transporter 8

## Supplementary Materials

The following are available online at https://www.mdpi.com/2073-4425/11/10/1221/s1, Figure S1: Frequency distribution of 149 F_2_ (S-91 × S-297) lines, Table S1: List of sesame core-collection genotypes, Table S2: ANOVA for the effect of continent on morphological, phenotypic and seed mineral nutrient concentrations among 30 sesame genotypes, Table S3: ANOVA for the effect of genotype on morphological, phenotypical and seed mineral nutrient concentrations among 30 sesame genotypes, Table S4: Coefficients of correlation (r) between morphological, phenotypic and seed mineral nutrient concentrations among 30 sesame genotypes, Table S5: Phenotypic and genotypic coefficients of correlation (r) between phenological, morphological and seed quality traits in S-91 × S-297 F_2_ population, Table S6: Mapping population recombination bin map containing 2339 bin markers, Table S7: Quantitative trait loci for plant phenological, morphological and seed quality traits, Table S8: Genes found within selected multi-traits QTL intervals, Table S9: Putative candidate genes selected from multi-traits QTL intervals, Table S10: Iron bio-accessibility in different food sources, Table S11: Mineral-nutrient bio-accessibility of sesame seed paste in the parental.

## References

[1] Gharibzahedi S.M.T., Jafari S.M. (2017). The importance of minerals in human nutrition: Bioavailability, food fortification, processing effects and nanoencapsulation. Trends Food Sci. Technol..

[2] Müller O., Krawinkel M. (2005). Malnutrition and health in developing countries. Can. Med. Assoc. J..

[3] Garg M., Sharma N., Sharma S., Kapoor P., Kumar A., Chunduri V., Arora P. (2018). Biofortified crops generated by breeding, agronomy, and transgenic approaches are improving lives of millions of people around the world. Front. Nutr..

[4] Platel K., Srinivasan K. (2015). Bioavailability of micronutrients from plant foods: An update. Crit. Rev. Food Sci. Nutr..

[5] White P.J., Broadley M.R. (2009). Biofortification of crops with seven mineral elements often lacking in human diets-iron, zinc, copper, calcium, magnesium, selenium and iodine. New Phytol..

[6] Lott J.N., Ockenden I., Raboy V., Batten G.D. (2000). Phytic acid and phosphorus in crop seeds and fruits: A global estimate. Seed Sci. Res..

[7] Gadri Y., Williams L.E., Peleg Z. (2020). Tradeoffs between yield components promote crop stability in sesame. Plant Sci..

[8] Çağırgan M.I. (2006). Selection and morphological characterization of induced determinate mutants in sesame. Field Crop. Res..

[9] Mushtaq A., Hanif M.A., Ayub M.A., Bhatti I.A., Jilani M.I., Hanif M.A., Nawaz H., Khan M.M., Byrne H.J. (2020). Sesame. Medicinal Plants of South Asia.

[10] Uzun B., Arslan Ç., Furat Ş. (2008). Variation in fatty acid compositions, oil content and oil yield in a germplasm collection of sesame (*Sesamum indicum* L.). J. Am. Oil Chem. Soc..

[11] Pathak N., Rai A.K., Kumari R., Thapa A., Bhat K.V. (2014). Sesame Crop: An Underexploited Oilseed Holds Tremendous Potential for Enhanced Food Value. Agric. Sci..

[12] Johnson L.A., Suleiman T.M., Lusas E.W. (1979). Sesame protein: A review and prospectus. J. Am. Oil Chem. Soc..

[13] Dimitrios B. (2006). Sources of natural phenolic antioxidants. Trends Food Sci. Technol..

[14] Bedigian D. (2011). Sesame: The genus Sesamum.

[15] Tripathy S.K., Kar J., Sahu D., Al-Khayri J.M., Jain S.M., Johnson D.V. (2019). Advances in sesame (*Sesamum indicum* L.) breeding. Advances in Plant Breeding Strategies: Industrial and Food Crops.

[16] Pham T.D., Geleta M., Bui T.M., Bui T.C., Merker A., Carlsson A.S. (2011). Comparative analysis of genetic diversity of sesame (*Sesamum indicum* L.) from Vietnam and Cambodia using agro-morphological and molecular markers. Hereditas.

[17] Basak M., Uzun B., Yol E. (2019). Genetic diversity and population structure of the Mediterranean sesame core collection with use of genome-wide SNPs developed by double digest RAD-Seq. PLoS ONE.

[18] Pandey S.K., Das A., Rai P., Dasgupta T. (2015). Morphological and genetic diversity assessment of sesame (*Sesamum indicum* L.) accessions differing in origin. Physiol. Mol. Biol. Plants.

[19] Whan A.P., Smith A.B., Cavanagh C.R., Ral J.-P.F., Shaw L.M., Howitt C.A., Bischof L. (2014). GrainScan: A low cost, fast method for grain size and colour measurements. Plant Methods.

[20] Minekus M., Alminger M., Alvito P., Ballance S., Bohn T., Bourlieu C., Carriere F., Boutrou R., Corredig M., Dupont D. (2014). A standardised static in vitro digestion method suitable for food–an international consensus. Food Funct..

[21] Doyle J., Doyle J. (1987). Genomic plant DNA preparation from fresh tissue-CTAB method. Phytochem. Bull..

[22] Wang L., Xia Q., Zhang Y., Zhu X., Zhu X., Li D., Ni X., Gao Y., Xiang H., Wei X. (2016). Updated sesame genome assembly and fine mapping of plant height and seed coat color QTLs using a new high-density genetic map. BMC Genom..

[23] Thorvaldsdóttir H., Robinson J.T., Mesirov J.P. (2012). Integrative Genomics Viewer (IGV): High-performance genomics data visualization and exploration. Brief. Bioinform..

[24] Peleg Z., Cakmak I., Ozturk L., Yazici A., Jun Y., Budak H., Korol A.B., Fahima T., Saranga Y. (2009). Quantitative trait loci conferring grain mineral nutrient concentrations in durum wheat × wild emmer wheat RIL population. Theor. Appl. Genet..

[25] Ragel P., Raddatz N., Leidi E.O., Quintero F.J., Pardo J.M. (2019). Regulation of K^+^ nutrition in plants. Front. Plant Sci..

[26] Khan G.A., Bouraine S., Wege S., Li Y., De Carbonnel M., Berthomieu P., Poirier Y., Rouached H. (2014). Coordination between zinc and phosphate homeostasis involves the transcription factor PHR1, the phosphate exporter PHO1, and its homologue PHO1;H3 in Arabidopsis. J. Exp. Bot..

[27] Connolly E.L., Campbell N.H., Grotz N., Prichard C.L., Guerinot M.L. (2003). Overexpression of the FRO2 ferric chelate reductase confers tolerance to growth on low iron and uncovers posttranscriptional control. Plant Physiol..

[28] Kaplan B., Sherman T., Fromm H. (2007). Cyclic nucleotide-gated channels in plants. FEBS Lett..

[29] Lee S., Kim S.A., Lee J., Guerinot M.L., An G. (2010). Zinc deficiency-inducible *OsZIP8* encodes a plasma membrane-localized zinc transporter in rice. Mol. Cells.

[30] Tulchinsky T.H. (2010). Micronutrient deficiency conditions: Global health issues. Public Heal. Rev..

[31] Furat S., Uzun B. (2010). The use of agro-morphological characters for the assessment of genetic diversity in sesame (*Sesamum indicum* L.). Plant Omics.

[32] Arriel N.H.C., Di Mauro A.O., Arriel E.F., Unêda-Trevisoli S.H., Costa M.M., Bárbaro I.M., Muniz F.R.S. (2007). Genetic divergence in sesame based on morphological and agronomic traits. Crop Breed. Appl. Biotechnol..

[33] Wei X., Liu K., Zhang Y., Feng Q., Wang L., Zhao Y., Li D., Zhao Q., Zhu X., Zhu X. (2015). Genetic discovery for oil production and quality in sesame. Nat. Commun..

[34] Cai G., Yang Q.-Y., Chen H., Yang Q., Zhang C., Fan C., Zhou Y. (2016). Genetic dissection of plant architecture and yield-related traits in Brassica napus. Sci. Rep..

[35] Langham D.R., Janick J., Whipkey A. (2007). Phenology of sesame. Issues in New Crops and New Uses.

[36] Zhao F., Su Y., Dunham S., Rakszegi M., Bedo Z., McGrath S., Shewry P. (2009). Variation in mineral micronutrient concentrations in grain of wheat lines of diverse origin. J. Cereal Sci..

[37] Menkir A. (2008). Genetic variation for grain mineral content in tropical-adapted maize inbred lines. Food Chem..

[38] Pinson S.R.M., Tarpley L., Yan W., Yeater K., Lahner B., Yakubova E., Huang X.-Y., Zhang M., Guerinot M.L., Salt D.E. (2015). Worldwide genetic diversity for mineral element concentrations in rice grain. Crop Sci..

[39] Mamo B.E., Barber B.L., Steffenson B.J. (2014). Genome-wide association mapping of zinc and iron concentration in barley landraces from Ethiopia and Eritrea. J. Cereal Sci..

[40] Chatzav M., Peleg Z., Ozturk L., Yazici A., Fahima T., Cakmak I., Saranga Y. (2010). Genetic diversity for grain nutrients in wild emmer wheat: Potential for wheat improvement. Ann. Bot..

[41] Garcia-Oliveira A.L., Tan L., Fu Y., Sun C. (2009). Genetic identification of quantitative trait loci for contents of mineral nutrients in rice grain. J. Integr. Plant Biol..

[42] Blair M.W., Astudillo C., Grusak M.A., Graham R., Beebe S.E. (2008). Inheritance of seed iron and zinc concentrations in common bean (*Phaseolus vulgaris* L.). Mol. Breed..

[43] Grotz N., Guerinot M.L. (2006). Molecular aspects of Cu, Fe and Zn homeostasis in plants. Biochim. Biophys. Acta BBA-Mol. Cell Res..

[44] Ajeesh Krishna T.P., Maharajan T., Victor Roch G., Ignacimuthu S., Ceasar S.A. (2020). Structure, function, regulation and phylogenetic relationship of ZIP family tansporters of plants. Front. Plant Sci..

[45] Šimić D., Sudar R., Ledenčan T., Jambrović A., Zdunić Z., Brkić I., Kovačević V. (2009). Genetic variation of bioavailable iron and zinc in grain of a maize population. J. Cereal Sci..

[46] Shunmugam A., Bock C., Arganosa G.C., Georges F., Gray G.R., Warkentin T.D. (2015). Accumulation of Phosphorus-containing compounds in developing seeds of low-phytate pea (*Pisum sativum* L.) mutants. Plants.

[47] Abebe Y., Bogale A., Hambidge K.M., Stoecker B.J., Bailey K., Gibson R.S. (2007). Phytate, zinc, iron and calcium content of selected raw and prepared foods consumed in rural Sidama, Southern Ethiopia, and implications for bioavailability. J. Food Compos. Anal..

[48] Cichy K.A., Caldas G.V., Snapp S.S., Blair M.W. (2009). QTL Analysis of Seed Iron, Zinc, and Phosphorus Levels in an Andean Bean Population. Crop Sci..

[49] Stangoulis J.C., Huynh B.-L., Welch R.M., Choi E.-Y., Graham R.D. (2007). Quantitative trait loci for phytate in rice grain and their relationship with grain micronutrient content. Euphytica.

[50] Zhang H., Miao H., Wei L., Li C., Duan Y., Xu F., Qu W., Zhao R., Ju M., Chang S. (2018). Identification of a SiCL1 gene controlling leaf curling and capsule indehiscence in sesame via cross-population association mapping and genomic variants screening. BMC Plant Biol..

[51] Gaymard F., Pilot G., Lacombe B., Bouchez D., Bruneau D., Boucherez J., Michaux-Ferrière N., Thibaud J.-B., Sentenac H. (1998). Identification and disruption of a plant shaker-like outward channel involved in K^+^ release into the xylem sap. Cell.

[52] Zelman A.K., Dawe A., Gehring C., Berkowitz G.A. (2012). Evolutionary and structural perspectives of plant cyclic nucleotide-gated cation channels. Front. Plant Sci..

[53] Guerinot M.L. (2000). The ZIP family of metal transporters. Biochim. Biophys. Acta BBA-Biomembr..

[54] Rossander-Hultén L., Brune M., Sandström B., Lönnerdal B., Hallberg L. (1991). Competitive inhibition of iron absorption by manganese and zinc in humans. Am. J. Clin. Nutr..

[55] Hallberg L., Hulthén L. (2000). Prediction of dietary iron absorption: An algorithm for calculating absorption and bioavailability of dietary iron. Am. J. Clin. Nutr..

[56] Seshadri S. (2001). Prevalence of micronutrient deficiency particularly of iron, zinc and folic acid in pregnant women in South East Asia. Br. J. Nutr..

[57] Scheers N. (2013). Regulatory Effects of Cu, Zn, and Ca on Fe absorption: The intricate play between nutrient transporters. Nutrients.

[58] Mulet-Cabero A.-I., Egger L., Portmann R., Ménard O., Marze S., Minekus M., Le Feunteun S., Sarkar A., Grundy M.M.L., Carrière F. (2020). A standardised semi-dynamic in vitro digestion method suitable for food-an international consensus. Food Funct..

