# Supplementary material for: Genetic Architecture Underpinning Yield Components and Seed Mineral–Nutrients in Sesame
Source: Genes (Basel). 2020 Oct 18;11(10):1221. doi: 10.3390/genes11101221 (PMC7603122; doi:10.3390/genes11101221)
Supplement: Supplementary file 1 [file genes-11-01221-s001.zip › SI Data/Teboul et al. SI Figures.pdf]

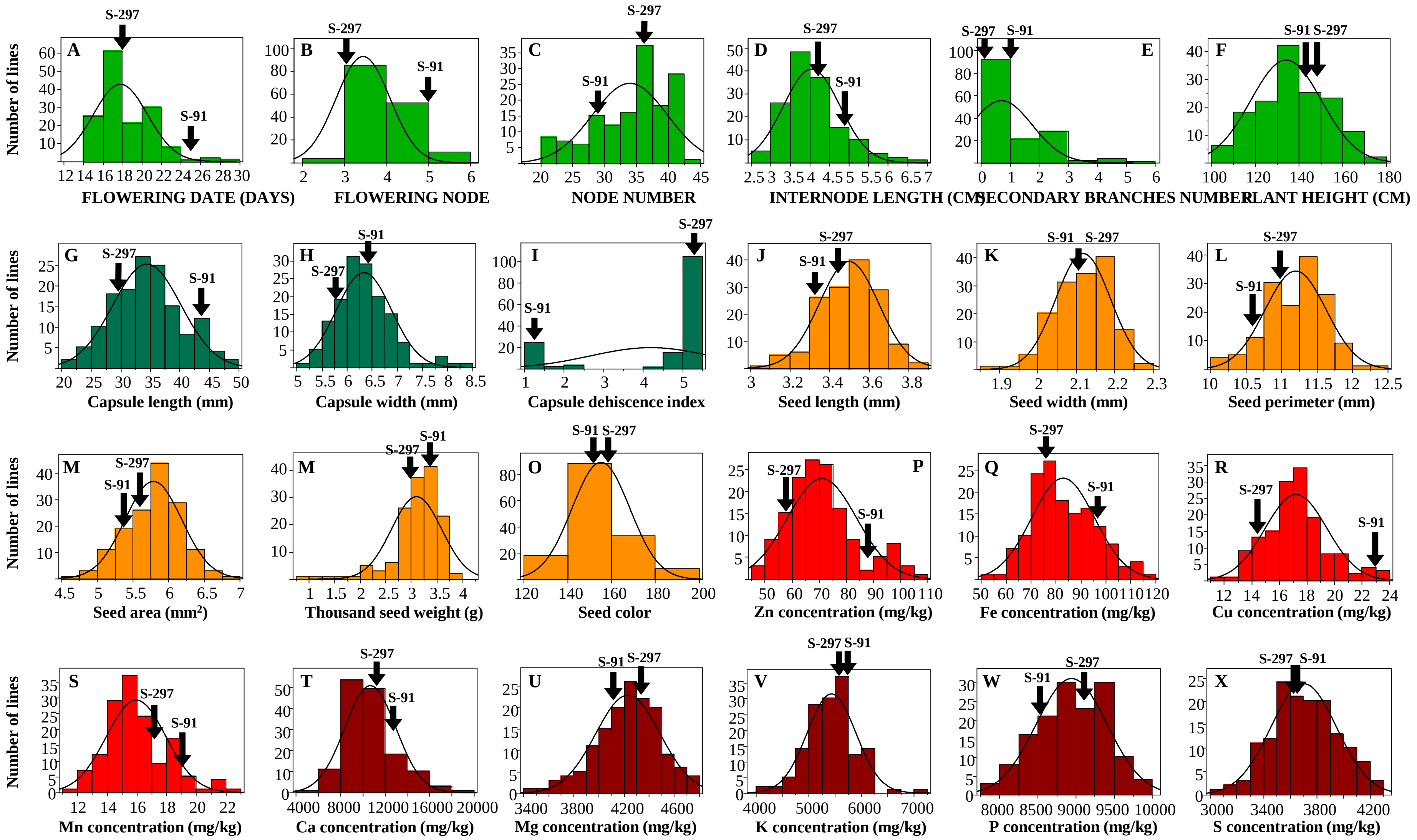

Figure S1. Frequency distribution of 149 F_2 (S-91 × S-297) lines for plant phenological and morphological traits (green): (**A**) flowering date, (**B**) flowering node, (**C**) node number, (**D**) internode length, (**E**) secondary branches number, and (**F**) plant height. Capsule morphology traits (dark green): (**G**) capsule length, (**H**) capsule width, (**I**) capsule dehiscence index, (**J**) seed length, (**K**) seed width, (**l**) seed perimeter, and (**M**) seed area. Seed quality traits (orange): (**N**) thousand seed weight, and (**O**) seed color. Seed concentrations micronutrients (red): (**P**) zinc, (**Q**) iron, (**R**) copper, (**S**) manganese, and macronutrients (burgundy): (**T**) calcium, (**U**) magnesium, (**V**) potassium, (**W**) phosphorus and (**X**) sulfur. S-91 and S-297 parental lines are indicated above with arrows.
